# A novel prognostic model for cutaneous melanoma based on an immune-related gene signature and clinical variables

**DOI:** 10.1038/s41598-022-23475-4

**Published:** 2022-11-27

**Authors:** Yifan Tang, Huicong Feng, Lupeng Zhang, Chiwen Qu, Jinlong Li, Xiangyu Deng, Suye Zhong, Jun Yang, Xiyun Deng, Xiaomin Zeng, Yiren Wang, Xiaoning Peng

**Affiliations:** 1 Department of Pathology and Pathophysiology, Hunan Normal University School of Medicine, Changsha, 410013 Hunan China; 2 Department of Biochemistry and Molecular Biology, Jishou University School of Medicine, Jishou, 416000 Hunan China; 3 Department of Statistics, College of Mathematics and Computer Science, Hunan Normal University, Changsha, 410081 Hunan China; 4 School of Public Health and Management, Youjiang Medical University for Nationalities, Baise, 533000 Guangxi China; 5 Department of Epidemiology and Health Statistics, Xiangya Public Health School, Central South University, Changsha, 410078 Hunan China

**Keywords:** Cancer, Computational biology and bioinformatics, Immunology

## Abstract

Abundant evidence has indicated that the prognosis of cutaneous melanoma (CM) patients is highly complicated by the tumour immune microenvironment. We retrieved the clinical data and gene expression data of CM patients in The Cancer Genome Atlas (TCGA) database for modelling and validation analysis. Based on single-sample gene set enrichment analysis (ssGSEA) and consensus clustering analysis, CM patients were classified into three immune level groups, and the differences in the tumour immune microenvironment and clinical characteristics were evaluated. Seven immune-related CM prognostic molecules, including three mRNAs (*SUCO*, *BTN3A1* and *TBC1D2*), three lncRNAs (*HLA-DQB1-AS1*, *C9orf139* and *C22orf34*) and one miRNA (*hsa*-*miR-17-5p*), were screened by differential expression analysis, ceRNA network analysis, LASSO Cox regression analysis and univariate Cox regression analysis. Their biological functions were mainly concentrated in the phospholipid metabolic process, transcription regulator complex, protein serine/threonine kinase activity and MAPK signalling pathway. We established a novel prognostic model for CM integrating clinical variables and immune molecules that showed promising predictive performance demonstrated by receiver operating characteristic curves (AUC ≥ 0.74), providing a scientific basis for predicting the prognosis and improving the clinical outcomes of CM patients.

## Introduction

Every year, there are more than 280,000 new patients and 60,000 deaths of cutaneous melanoma (CM) worldwide^1^. GLOBOCAN 2020 estimates that 324,635 new CM cases and 57,043 new CM deaths occurred in 2020^2,3^. Four subtypes of CM, namely superficial spreading melanoma, nodular melanomas, lentigo maligna melanoma and acral lentiginous melanoma, have been identified pathologically^4,5^. CM is highly malignant, is prone to lymph node and haematogenous metastases at the early stage of the disease, and has a poor clinical prognosis. The median survival time of CM patients in stage IV is only 4–9 months, and the 3-year survival rate of stage IV patients is less than 20%^6,7^. The 5-year survival rates of CM patients are 97% forstage IA, 84% for stage IB, 68% for stage II, 55% for stage III, and 17% for stage IV^7^. The clinical diagnosis is easily affected by subjective factors, the histological grade cannot fully reflect the biological behaviour of CM. Increasing evidence shows that the prognosis of CM is related to molecular abnormalities, such as mutant *P53*, *BRAF*, *KIT*, *NF1*, *RUNX3*, *S100*, *VEGF*, *LDH* and *MIA*, which are involved in the occurrence, development, invasion and metastasis of CM^8–13^. Therefore, it is difficult to accurately predict the prognosis of CM solely based on clinical variables.

The tumour microenvironment is composed of cancer cells, surrounding blood vessels, infiltrating immune cells, fibroblasts, signalling molecules and the extracellular matrix^14^. The tumour microenvironment affects the gene expression of tumour tissue in a variety of ways, allowing tumour cells to escape immunity and then affecting the occurrence, development and therapeutic efficacy of the tumour^15–17^. Studies have shown that immune system components are associated with the occurrence and development of CM^18^. For example, immune cell infiltration is an effective prognostic factor of CM^19,20^. However, new immunotherapies based on immune checkpoints benefit only a few melanoma patients^21^. Therefore, it is necessary to screen the molecular prognostic indicators of the CM immune microenvironment and establish a prognostic prediction model integrating clinical and immune characteristics, which will help to improve the accuracy of prognosis prediction and targeted therapy of CM patients.

In this study, we used the gene expression data of CM patients in TCGA (The Cancer Genome Atlas) to screen CM prognostic molecules using the least absolute shrinkage and selection operator (LASSO) Cox regression algorithm. A prognostic model of CM integrating with clinical variables and immune characteristics was established by multivariable Cox regression, providing a scientific basis for exploring the prognostic prediction of CM and improving the clinical treatment of CM patients.

## Results

### Clinical characteristics of CM patients

**Figure 1:**
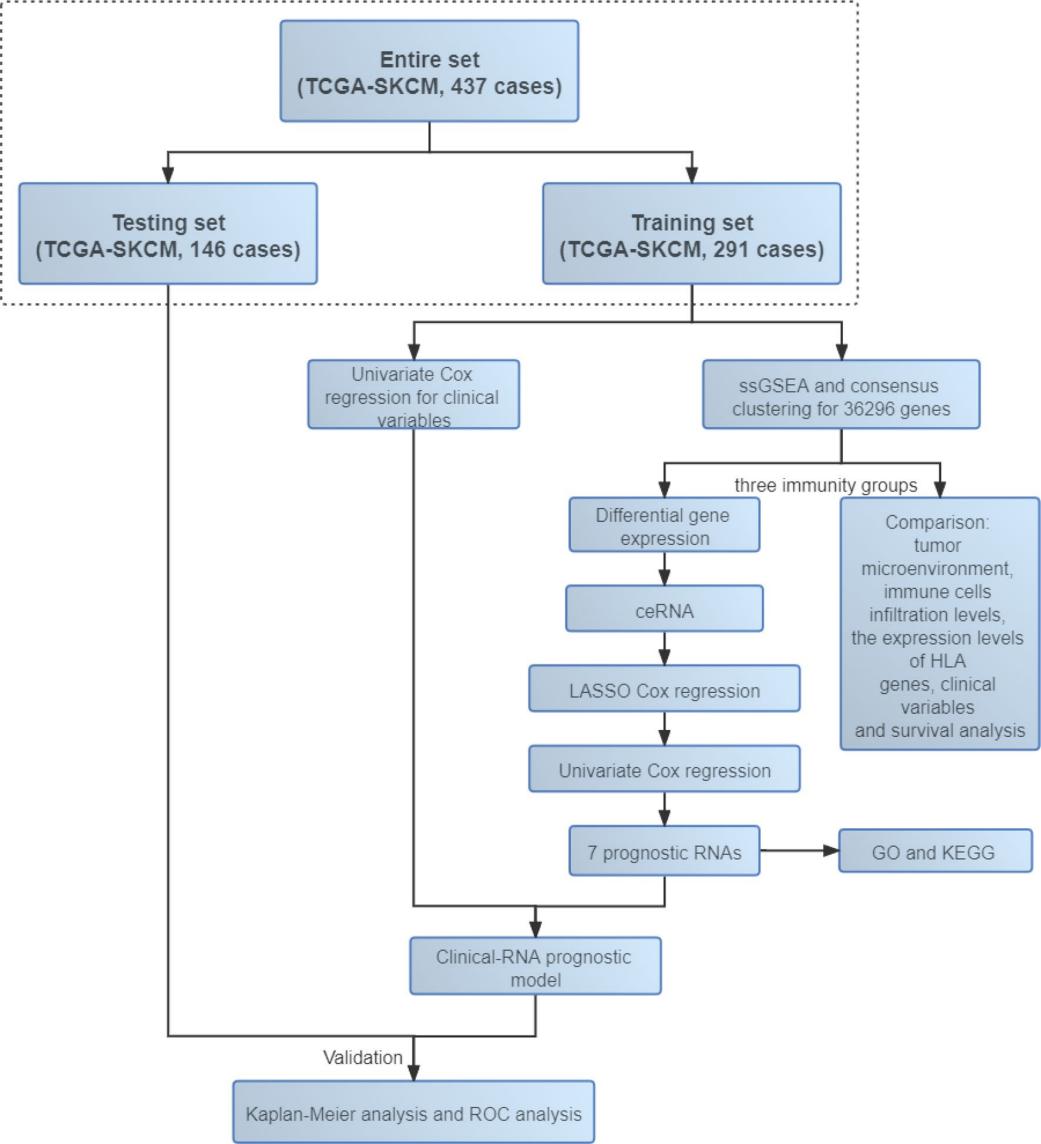
Brief flow chart of this study.

The flow chart of this study is shown in Fig. 1. In the training set of 291 CM patients, the longest survival time was 30.73 years and the median survival time was 7.75 years. The average age at initial pathological diagnosis of CM was 57.33 ± 16.35 (years). There were 275 white CM patients (97.17%), 261 primary neoplasm cases (89.68%), 269 patients with CM without systemic treatment (92.44%), 139 CM patients with local lymph node infiltration (48.26%) and 105 patients (39.77%) with tumours at stage III. The clinical information is shown in Table 1.

**Table 1:** Clinical characteristics and univariate Cox regression of overall survival and immune level grouping results of CM patients in the training set.

Clinical variables	Training set (n = 291)	Univariate Cox regression		Immune level groups			p-value
		HR (95% CI)	p-value	Immunity-H (n = 98)	Immunity-M (n = 82)	Immunity-L (n = 111)	
Age at initial diagnosis (years, mean ± SD)	57.33 ± 16.35	1.025 (1.014–1.036)	< 0.001	56.67 ± 16.08	58.62 ± 15.12	56.96 ± 17.51	0.696
**Gender (n, %)**		0.868 (0.610–1.236)	0.432				0.070
Male	181 (62.20)			53 (53.06)	54 (65.85)	75 (67.57)	
Female	110 (37.80)			46 (46.94)	28 (34.15)	36 (32.43)	
**Race (n, %)**^**c**^		1.331 (0.714–2.481)	0.369				0.960
White	275 (97.17)			93 (96.88)	77 (97.47)	104 (97.20)	
Asian	7 (2.48)			2 (2.08)	2 (2.53)	3 (2.80)	
Black or African American	1 (0.35)			1 (1.04)	0 (0.00)	0 (0.00)	
Not reported	8						
**Pathologic-M**		1.604 (0.745–3.456)	0.227				0.115
M0	253 (92.67)			89 (96.74)	70 (88.61)	94 (93.07)	
M1	20 (7.33)			3 (3.26)	9 (11.39)	7 (6.93)	
Not reported	19						
**Pathologic-N**		1.293 (1.099–1.522)	0.002				0.260
N0	148 (56.92)			49 (55.68)	42 (56.00)	57 (58.76)	
N1	45 (17.32)			11 (12.50)	14 (18.67)	20 (20.62)	
N2	27 (10.38)			10 (11.36)	10 (13.33)	7 (7.22)	
N3	40 (15.38)			18 (20.45)	9 (12.00)	13 (13.40)	
NX	17						
Not reported	14						
**Pathologic-T**		1.455 (1.223–1.732)	< 0.001				0.001
T0	14 (5.88)			8 (10.39)	3 (4.17)	3 (3.37)	
T1	29 (12.18)			17 (22.08)	8 (11.11)	4 (4.49)	
T2	47 (19.75)			15 (19.48)	18 (25.00)	14 (15.73)	
T3	55 (23.11)			17 (22.08)	13 (18.06)	25 (28.09)	
T4	93 (39.08)			20 (25.97)	30 (41.67)	43 (48.31)	
Tis	6						
TX	27						
Not reported	20						
**Primary neoplasm (n, %)**		0.740 (0.375–1.460)	0.385				0.134
Yes	261 (89.69)			83 (84.69)	76 (92.68)	102 (91.89)	
No	30 (10.31)			15 (15.31)	6 (7.32)	9 (8.11)	
**Previous systemic treatment**		1.073 (0.627–1.837)	0.797				0.010
Yes	22 (7.56)			3 (3.06)	4 (4.88)	15 (13.51)	
No	269 (92.44)			95 (96.94)	78 (95.12)	96 (86.49)	
**Primary radiotherapy**		1.271 (0.403–4.003)	0.682				0.353
Yes	8 (3.15)			1 (1.25)	2 (2.70)	5 (5.00)	
No	246 (96.85)			79 (98.75)	72 (97.30)	95 (95.00)	
Not reported	37						
**Tumor location**^**a**^		0.916 (0.803–1.044)	0.189				< 0.001
Distant metastasis	46 (15.97)			6 (6.19)	10 (12.20)	30 (27.52)	
Primary tumor	59 (20.49)			12 (12.37)	20 (24.39)	27 (24.77)	
Regional cutaneous or subcutaneous tissue^b^	44 (15.28)			10 (10.31)	18 (21.95)	16 (14.68)	
Regional lymph node	139 (48.26)			69 (71.13)	34 (41.46)	36 (33.03)	
Not reported	3						
**Tumor stage (n, %)**		1.344 (1.109–1.630)	0.003				0.499
0	6 (2.27)			2 (2.33)	0 (0.00)	4 (3.96)	
I	49 + 3 (19.70)			25 (29.07)	16 (20.78)	11 (10.89)	
II	81 + 2 (31.44)			18 (20.93)	24 (31.17)	41 (40.59)	
III	105 (39.77)			38 (44.19)	29 (37.66)	38 (37.62)	
IV	18 (6.82)			3 (3.49)	8 (10.39)	7 (6.93)	
I/II	5^c^						
Not reported	27						

*HR* hazard ratio, *95% CI* 95% confidence interval, *SD* standard deviation.

^a^Dummy variable.

^b^Includes satellite and in-transit metastasis.

^c^The 5 CM patients were randomly assigned to I (n = 3) or II (n = 2).

### Grouping of CM patients based on the immune level

**Figure 2:**
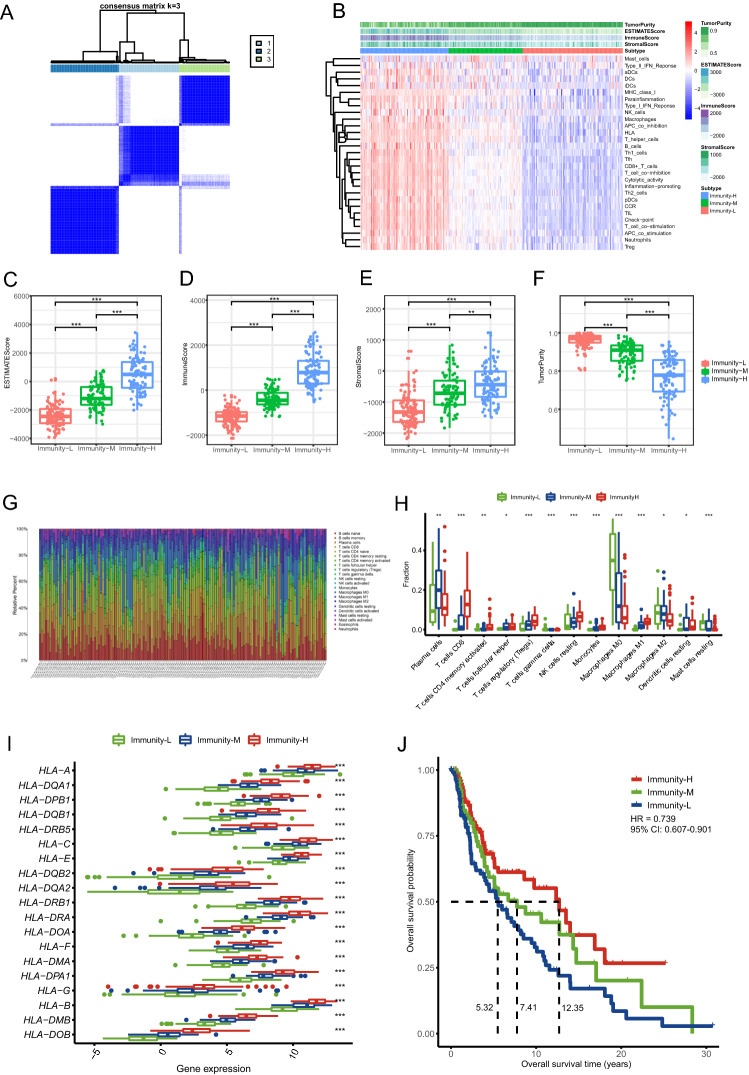
Three immune level groups were determined by ssGSEA and consensus clustering analysis. Cluster process diagram of consensus clustering analysis (**A)**. Heatmap of the ssGSEA score for all CM patients in the training set (**B)**. ESTIMATE score (**C)**, immune score (**D)**, stromal score (**E),** and tumor purity (**F)** of the different immune groups. The bar graph shows the proportion of 22 kinds of immune cells in CM tumor samples in the training set. Each column indicates one sample (**G)**. The box plot shows the differentiation of 13 kinds of immune cell fraction within the different immune groups (**H)**. The box plot displays the difference in HLA gene expression within the different immune groups (**I)**. Kaplan–Meier curves of the three different immune groups and the result of univariate Cox analysis. HR, hazard ratio. 95% CI, 95% confidence interval (**J)**. *p < 0.05, **p < 0.01, ***p < 0.001.

The single-sample gene set enrichment analysis (ssGSEA) score calculated by the analysis of 29 immune-related gene sets of each CM patient in the training set was obtained to quantify the activity or enrichment levels of immune cells, functions, or pathways (Table S1). We consensually clustered 291 CM patients in the training set into three immune level groups, and defined these groups as: Immunity-H (n = 98), Immunity-M (n = 82) and Immunity-L (n = 111) (Fig. 2A,B; Table 1). We found that the ESTIMATE score, immune score, stromal score and tumour purity were significantly different among the three groups (p < 0.05). The ESTIMATE score, immune score and stromal score of the Immunity-H group were the highest, and the tumour purity was the lowest; the Immune-L group had the lowest ESTIMATE score, immune score and stromal score, and the highest tumour purity (Fig. 2C–F).

The analysis of the relative proportion of immune cells showed that the infiltration levels of 13 kinds of immune cells had significant differences among the three immune groups (p < 0.05). Among them, CD8^+^ T cells, CD4^+^ memory activated T cells, follicular helper T cells, regulatory T cells (Tregs), gamma delta T cells, resting NK cells, monocytes, M1 macrophages and resting dendritic cells were more abundantly infiltrated in the Immunity-H group (Fig. 2G,H).

We then examined the HLA gene expression levels and found that the expression of the HLA gene family had significant differences among the three immune groups (p < 0.05). Most HLA genes had the highest expression levels in the Immunity-H group and the lowest expression levels in the Immunity-L group (Fig. 2I).

The comparison of clinical variables among the three immune groups showed that there were differences in pathologic-T stage, prior systemic therapy and tumour location among the three groups (p < 0.05) (Table 1). The numbers of CM patients with pathologic-T (T3 + T4) stage in the Immunity-H group, Immunity-M group, and Immunity-L group were 37 (48.05%), 43 (59.73%), and 68 (76.40%), respectively, suggesting that the range or degree of tumour involvement in the Immunity-L group was the highest. The number of CM patients with prior systemic therapy was the lowest in the Immunity-H group (3/291 = 3.06%) and the highest in the Immunity-L group (15/291 = 13.51%). The numbers of patients with distant metastasis in the Immunity-H group, Immunity-M group, and Immunity-L group were 6 (6.19%), 10 (12.20%), and 30 (27.52%), respectively; the numbers of patients with tumour lymph node infiltration in the three groups were 69 (71.13%), 34 (41.46%) and 36 (33.03%).

The median survival times of CM patients in the Immunity-H group, Immunity-M group, and Immunity-L group were 12.35, 7.41, and 5.32 years, respectively. There were differences in overall survival (OS) among the three immune groups (HR = 0.739, 95% CI: 0.607–0.901). The patients in the Immunity-H group had the best prognosis, while those in the Immunity-L group had the worst prognosis (Fig. 2J).

### Immune molecules related to CM prognosis

**Figure 3:**
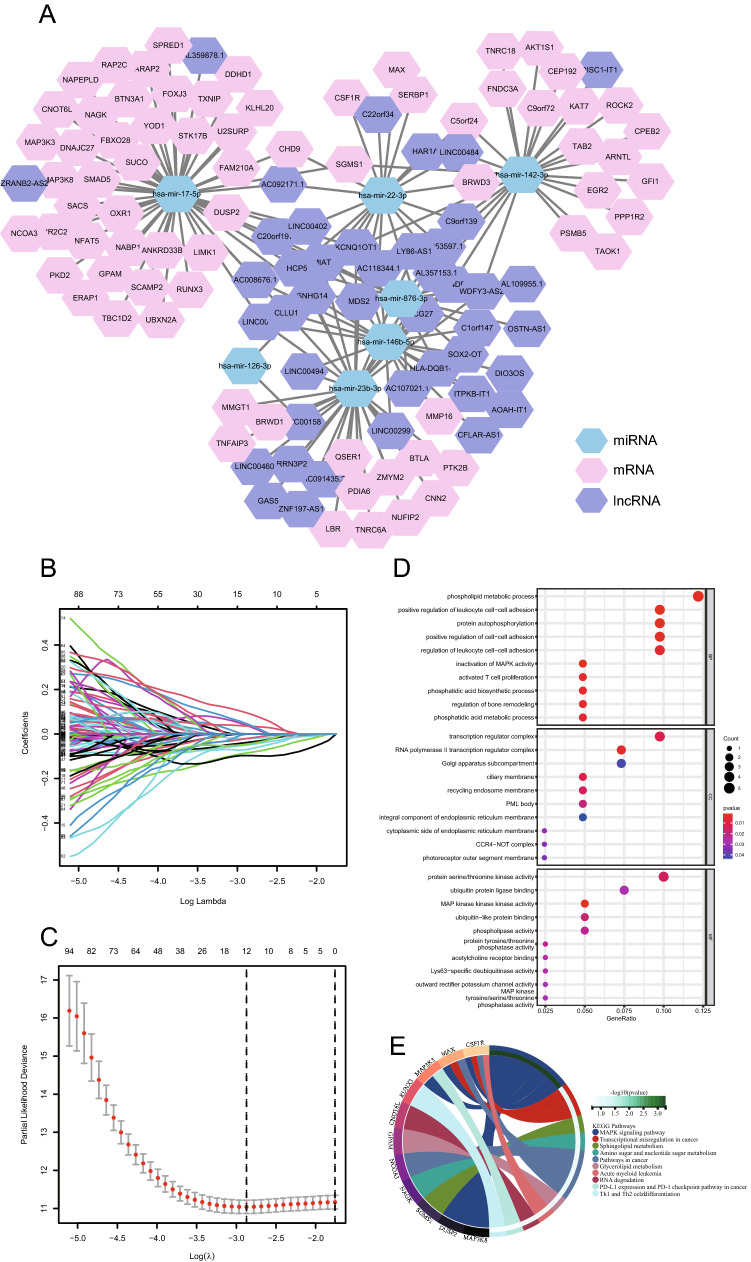
The immune molecules related to the prognosis of CM were screened. A ceRNA network of the differentially expressed lncRNAs, miRNAs and mRNAs in different immune groups (**A)**. LASSO coefficient profiles of the genes in the ceRNA network (**B)**. A coefficient profile plot was generated against the log (λ) sequence. Selection of the optimal parameter (λ) in the LASSO Cox regression analysis (**C)**. Enrichment of top 10 GO terms (p ≤ 0.05) (**D)** and KEGG pathways (**E)** of the mRNAs associated with the seven selected immune molecules related to the prognosis of CM patients.

The Kruskal‒Wallis test was applied to analyse the gene expression levels of the three immune groups in the training set. The numbers of differentially expressed mRNAs, lncRNAs and miRNAs were 6,807, 1,828, and 255, respectively (p < 0.05) (Table S2). Based on these differentially expressed RNAs, we obtained 245 miRNA‒lncRNA pairs, 198 miRNA‒mRNA pairs and 224 lncRNA‒mRNA pairs. Then, we used the obtained RNA pairs to construct a ceRNA network composed of 72 mRNAs, 43 lncRNAs and 7 miRNAs (including 75 miRNA‒mRNA pairs and 89 miRNA‒lncRNA pairs) (Fig. 3A).

LASSO Cox regression was applied to screen CM prognostic RNAs from the RNAs in the ceRNA network, and eight RNAs were identified. The eight RNAs included four mRNAs (*SUCO*, *ANKRD33B*, *BTN3A1* and *TBC1D2*), three lncRNAs (*HLA-DQB1-AS1*, *C9orf139* and *C22orf34*) and one miRNA (*hsa*-*miR-17-5p*) (Table 2; Fig. 3B,C). The results of the univariate Cox regression for the eight RNAs showed that except for *ANKRD33B* (p = 0.595), the other seven RNAs were associated with the overall survival of CM patients (p < 0.05). These seven RNA molecules are immune molecules connected with the prognosis of CM patients. Among the seven statistically significant RNAs, the higher the expression levels of *hsa-miR-17-5p* and *TBC1D2* were, the greater the risk of death of CM patients (HR > 1, p < 0.05); the higher the expression levels of *HLA-DQB1-AS1*, *C9orf139*, *C22orf34*, *SUCO* and *BTN3A1* were, the lower the risk of death of CM patients (HR < 1, p < 0.05; Table 2). *TBC1D2*, *BTN3A1* and *SUCO* are the target genes of *hsa-miR-17-5p* (Fig. 3A).

**Table 2:** CM prognosis-related immune molecules screened by LASSO Cox regression and univariate Cox regression.

Gene symbol	Biotype	LASSO Cox regression coefficient	Univariate Cox regression	
			HR (95% CI)	p-value
*hsa-miR-17-5p*	MiRNA	0.156	1.154 (1.019–1.307)	0.024
*HLA-DQB1-AS1*	LncRNA	−0.120	0.864 (0.803–0.930)	< 0.001
*C9orf139*	lncRNA	−0.176	0.800 (0.719–0.891)	< 0.001
*C22orf34*	lncRNA	−0.103	0.885 (0.810–0.968)	0.007
*SUCO*	mRNA	−0.185	0.737 (0.601–0.903)	0.003
*ANKRD33B*	mRNA	0.187	1.024 (0.937–1.120)	0.595
*BTN3A1*	mRNA	−0.172	0.708 (0.602–0.834)	< 0.001
*TBC1D2*	mRNA	0.201	1.173 (1.002–1.372)	0.047

*HR* hazard ratio, *95% CI* 95% confidence interval.

The results of GO analysis of mRNAs related to the ceRNA network showed that these mRNAs were significantly enriched in biological processes related to phospholipid metabolism, positive regulation of leukocyte adhesion and protein autophosphorylation, cellular components related to the translation regulatory complex, RNA polymerase II transcription regulatory complex and Golgi subunit, and the molecular functions related to protein serine/threonine kinase, ubiquitin like protein binding and MAP kinase activity (p ≤ 0.05; Fig. 3D). The results of KEGG pathway enrichment analysis indicated that the CM prognostic molecules were significantly enriched in the MAPK signalling pathway (p ≤ 0.05; Fig. 3E).

### CM prognostic model combining clinical variables and immune molecules

**Figure 4:**
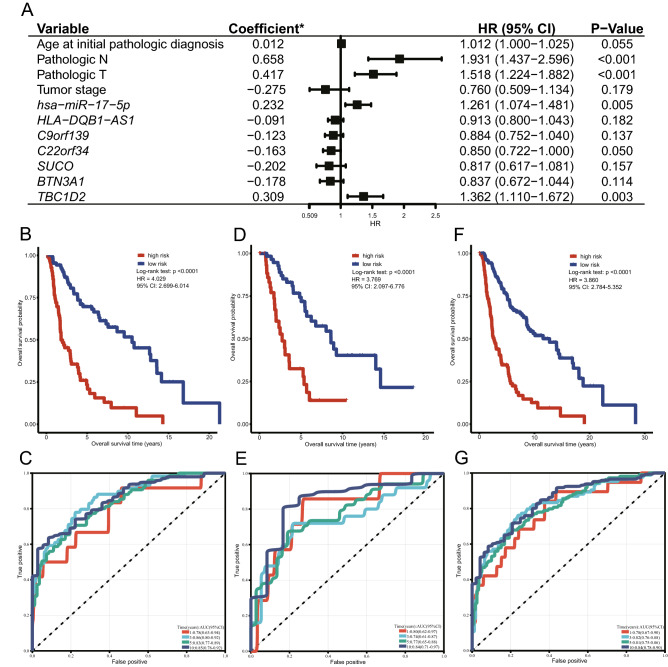
A CM prognosis model integrating CM prognosis related immune molecules and clinical variables. Forest plot of multivariate Cox regression for the integrated prognosis model (**A)**. Kaplan–Meier survival analyses for the integrated prognosis model based on the overall survival of the training set (**B)**, the testing set (**D),** and the entire set (**F)**, respectively. ROC analyses for the integrated prognosis model based on the overall survival of the training set (**C)**, the testing set (**E),** and the entire set (**G)**, respectively. Asterisk: multivariate Cox regression coefficient. *HR* hazard ratio, *95% CI* 95% confidence interval.

The results of univariate Cox regression showed that the clinical variables correlated with the prognosis of CM (p < 0.05) were age at initial diagnosis, pathologic-N stage, pathologic-T stage, and tumour stage (Table 1). The four clinical variables with statistical significance (p < 0.05) in univariate Cox regression and the seven RNA molecules related to the OS of the CM patients screened in this study were used as covariates for multivariate Cox regression analysis to fit the integrated prognostic model of clinical variable/immune molecules (mRNA/miRNA/lncRNA). The results showed that *hsa-miR-17-5p*, *C22orf34* and *TBC1D2* were independent prognostic factors of CM (Fig. 4A).

The predictive performance of the integrated clinical variables and immune molecules model was evaluated in the training set, the testing set and the entire set of data. The training set patients were stratified into a high-risk group and a low-risk group according to a cut-off value (median risk score) of 0.972. As depicted in the Kaplan–Meier survival curve, patients with higher risk scores had worse clinical outcomes (shorter OS time) than those with lower risk scores (HR = 4.029, p < 0.05). The ROC curves of the training set (AUC ≥ 0.78) demonstrated that the prognostic model had a promising ability to predict the prognostic risk of CM patients (Fig. 4B,C). This prognostic predictive accuracy of the integrated prognostic model was confirmed in the testing set (AUC ≥ 0.74; Fig. 4D,E) and the entire set (AUC ≥ 0.78; Fig. 4F,G).

## Discussion

**Figure 5:**
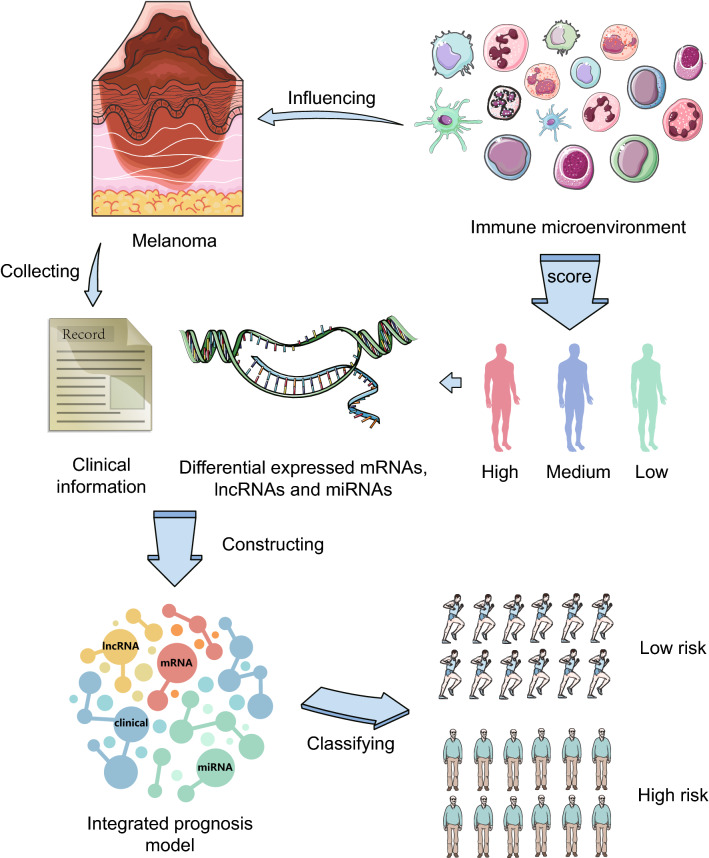
Graphical abstract. Tang et al. develop an immune-related method to predict the survival status of cutaneous melanoma patients. Comprehensive analysis of the nucleic acid and clinical information of the patients can obtain different prognostic groups. These factors may be the key to the treatment of cutaneous melanoma.

Melanoma with a large amount of immune cell infiltration is considered to be one of the most immunogenic tumours because of its high mutation load. Based on the clinical information of CM patients and the genome expression data of mRNAs, miRNAs and lncRNAs as a whole, we analysed the immune cell infiltration pattern of CM and identified seven immune-related CM prognostic RNA molecules, including three mRNAs, three lncRNAs and one miRNA, as well as four clinical variables related to CM prognosis. Based on these seven immune molecules and four clinical variables, an integrated prognostic model of clinical variables and mRNAs/miRNAs/lncRNAs was established. The AUC values of this prognostic model at 1-, 3-, 5- and 10 years were ≥ 0.74, revealing that the integrated prognostic model exhibited a promising predictive ability for the prognosis of CM patients (Fig. 5).

In this study, we screened three immune-related CM prognostic mRNAs: *SUCO*, *BTN3A1* and *TBC1D2*. The hazard ratios (HRs) of *SUCO* and *BTN3A1* were < 1, indicating that their increased expression led to a reduced risk of death in CM patients and that they are tumour suppressor genes in CM. *TBC1D2* was also an independent prognostic factor of CM, and CM patients with elevated expression levels of *TBC1D2* had an increased risk of death (HR > 1). Thus, *TBC1D2* acts as an oncogene in CM. *SUCO* is overexpressed in hepatocellular carcinoma (HCC) tissues, andhigh *SUCO* expression is significantly correlated with a low overall survival rate in HCC patients. *SUCO* may be a potential diagnostic biomarker in patients with liver cancer, promoting the occurrence and development of liver cancer as a tumour promoter^22^. The relationship between *SUCO* and CM has not been reported. The results of this study illustrate that *SUCO* may be a tumour suppressor gene in CM, suggesting that *SUCO* plays different roles in the occurrence and development of different tumours. *BTN3A1* belongs to the BTN3A family, which is part of a type I transmembrane protein of the immunoglobulin (Ig) superfamily. The expression levels of BTN3A family members are different among various tumours, which has a crucial impact on tumour prognosis^23^. In patients with melanoma, the expression of *BTN3A* is increased in pDC cells and γδ T cells; dysfunctional *BTN3A* leads to defective interaction between pDC and γδ T cells, affecting clinical results^24^. *TBC1D2* is a GTPase activating protein of Rab7, and *TBC1D2* contributes to the survival and proliferation of nasopharyngeal carcinoma cells and the metastasis of lung cancer cells^25,26^. The relationship between *TBC1D2* and melanoma has not been reported.

The three immune-related lncRNAs associated with CM prognosis were *HLA-DQB1-AS1*, *C9orf139* and *C22orf34*, and the results suggested that they play the roles of tumour suppressor genes and prognostic protective factors (HR < 1). *C22orf34* is an independent prognostic factor of CM. *HLA-DQB1-AS1* is considered to be a protective factor related to the prognosis of melanoma^27^, which is consistent with our findings. The lncRNA *C9orf139* is highly expressed in pancreatic cancer and can be used as a potential diagnostic and prognostic marker for pancreatic cancer. It promotes the growth of pancreatic cancer cells mediated by the *miR-663a/Sox12* axis^28^, playing a role as a tumour driver. The relationship between *C9orf139* and melanoma has not been reported. Whole genome sequencing revealed that *C22orf34* is a risk predictor of drug-induced interstitial lung disease^29^, and the relationship between *C22orf34* and melanoma has not been reported. The results of this study show that *C9orf139* and *C22orf34* may be tumour suppressor genes of CM, suggesting that *C9orf139* and *C22orf34* play different roles in different tumours.

One immune-related miRNA associated with CM prognosis was *hsa-miR-17-5p*, which was an independent prognostic factor of CM. Highly expressed *hsa-miR-17-5p* in CM plays the role of a tumour suppressor and prognostic risk factor (HR > 1). *Hsa-miR-17-5p* is a core member of the miR-17–92 family and is considered to be an oncogene. It promotes the malignant phenotype of breast cancer, prostate cancer and digestive system tumours^30–32^. *Hsa-miR-17-5p* enhances melanoma cell proliferation by targeting the ADAR1 protein^33^. This is consistent with our findings.

## Conclusion

This study found seven immune-related RNAs associated with the prognosis of CM patients, and established a clinical variable/immune molecule integrated prognostic model of CM with good predictive power. The novel model provides a scientific basis for discovering new prognostic markers of CM, clarifying the molecular mechanism of CM prognosis, and improving the prognosis and clinical management of CM patients.

## Materials and methods

### Data acquisition

The CM cohort data downloaded from the TCGA data portal included clinical, RNA-seq and miRNA-seq data (https://portal.gdc.cancer.gov/). The downloaded clinical dataset contains the clinical information of 470 CM patients, including age at initial pathologic diagnosis, gender, race, pathological classification, primary neoplasm, previous systemic therapy, primary radiotherapy, tumour location, tumour stage, survival status and survival time of CM patients. We omitted 10 of the 470 CM patients due to missing survival time information. The expression data (count) of RNA molecules were normalized and transformed to log_2_ (counts per million) with the “limma” R package^34^. Of the 460 CM patients, 437 patients had lncRNA, mRNA and miRNA expression data and survival data. The data of these 437 CM patients were included in this study for analysis. The 437 CM patients were randomly divided into a training set (n = 291) and a testing set (n = 146). The immune-related prognostic molecules of CM were screened in the training set, and the CM prognostic model was constructed. The testing set and entire dataset (n = 437) were used to verify the predictive ability of the model.

### Immune level grouping

Using the “limma”, “GSEABase” and “GSVA” R packages, we quantified the enrichment levels of the 29 immune-related gene sets in each CM patient (n = 291) of the training set by ssGSEA^35,36^. Based on the enrichment levels (ssGSEA scores) of the 29 immune signatures, we performed consensus clustering of the training set. There were 291 CM patients in the training set, which were divided into three groups with different immune levels, namely Immunity High (Immunity-H), Immunity Medium (Immunity-M) and Immunity Low (Immunity-L)^37^. The tumour microenvironment (TME) score of each patient was obtained with the “estimate” R package^38^. The differences in TME scores among the three immune groups were compared with the “ggpubr” R package. The CIBERSORT algorithm was used to explore the infiltration levels of 22 kinds of immune cells in melanoma tissue samples^39^, and the Kruskal‒Wallis test was used to compare the content of various types of immune cells among the three immune groups. The differences in HLA gene expression levels among the three immune groups were compared by one-way ANOVA. One-way ANOVA, the Kruskal‒Wallis test, the chi-square test and Fisher's exact test were used to compare the differences in clinical parameters among the three immune groups. Univariate Cox regression was used to evaluate the effect of the immune level on the overall survival of CM patients.

### Screening of immune-related prognostic molecules of CM

The Kruskal‒Wallis test with p values adjusted by Benjamini & Hochberg (BH) correction was used to compare the gene expression levels among the three immune groups, and the differentially expressed mRNAs, lncRNAs and miRNAs were screened in the training set. For these differentially expressed RNAs (p < 0.05), the miRcode database was used to obtain the relationship between miRNAs and lncRNAs; the target genes (mRNAs) of miRNAs were obtained through the miRDB, miRTarBase and TargetScan databases. These target genes (mRNAs) of miRNAs were intersected with differentially expressed mRNAs to obtain miRNA‒mRNA pairs; the lncRNA‒mRNA pairs were obtained by co-expression analysis of lncRNAs and RNAs (Pearson correlation coefficient > 0.4 and p < 0.001)^40^. The intersection of RNAs in miRNA‒lncRNA, miRNA‒mRNA and lncRNA‒mRNA pairs was used to construct the ceRNA network and to plot the ceRNA network diagram of lncRNA‒miRNA‒mRNA by Cytoscape^41^.

The LASSO Cox regression model of RNAs in the ceRNA network was constructed by the “glmnet” R package^42^. For each RNA molecule in the LASSO Cox regression model, the “survival” R package was used for univariate Cox regression analysis. RNA molecules with p ≤ 0.05 were identified as immune-related prognostic molecules of CM. The selected mRNAs associated with CM prognosis, the mRNAs appearing in the ceRNA network and the target mRNAs of the screened miRNAs and lncRNAs related to CM prognosis were analysed by Gene Ontology (GO) and Kyoto Encyclopedia of Genes and Genomes (KEGG) analyses using the “clusterProfiler” R package^43–45^. GO analysis mainly annotates and classifies genes through the biological process (BP), molecular function (MF), and cellular component (CC) categories to determine the shared functions of mRNAs (the enrichment threshold was p ≤ 0.05). KEGG analysis was performed to identify the signalling pathway in the enriched mRNAs (the threshold of enrichment was p ≤ 0.05).

### CM prognostic model integrating clinical variables and immune-related molecules

Univariate Cox regression analysis for clinical factors in the training set was implemented to identify the predictors of CM prognosis (p ≤ 0.05). Taking the screened prognostic RNA molecules of CM and the clinical variables affecting the prognosis of CM as covariates, the multivariate Cox regression model of CM was fitted to obtain the integrated prognostic model of clinical variables of CM and immune-related mRNAs, miRNAs and lncRNAs. Then, the prognostic value of the integrated model of clinical variables and mRNA/miRNA/lncRNA molecules was evaluated in the training set, the testing set, and the entire set. The risk score of CM patients was calculated as follows: ${\mathrm{riskscore}=\sum \mathrm{coef}}_{i}\times {X}_{i}$, where ${X}_{i}$ is the value of the variable in the integrated model and ${\mathrm{coef}}_{i}$ is the regression coefficient of the variable. The greater the risk score is, the worse the prognosis. Taking the median risk score of the training set as the cut-off value, the CM patients were divided into a high-risk group and a low-risk group, and the survival curves of the high-risk group and the low-risk group were drawn^46^. Univariate Cox regression and Kaplan‒Meier analysis with the log-rank test were used to compare the difference in the overall survival probability between the high-risk group and the low-risk group. The time-dependent receiver operating characteristic (ROC) curve was plotted using the “pROC” R package, and the time-dependent ROC curve and the area under the curve were obtained to evaluate the predictive performance of the prognostic model for CM patients. When the AUC reached 0.7, the prediction value was better.

### Statistics

All statistical calculations in this study were performed using R (version 4.0.5) software^47^. The significance level was α = 0.05 for univariate Cox regression analysis, multivariate Cox regression analysis, the log-rank test, one-way ANOVA, the Kruskal‒Wallis test, the chi-square test and Fisher's exact test.

### Ethics approval and consent to participate

Not applicable. This study only conducts data mining on public databases and does not involve any animal and clinical experiments. All methods were performed in accordance with the relevant guidelines and regulations.

## Supplementary Information

Supplementary Table S1.

Supplementary Table S2.

## Data Availability

The datasets generated and/or analyzed during the current study are available in the public open platform, including TCGA (https://portal.gdc.cancer.gov/), miRcode (http://www.mircode.org/), miRTarBase (http://mirtarbase.mbc.nctu.edu.tw/), miRDB (http://www.mirdb.org/) and TargetScan (http://www.targetscan.org/).The CM clinical data set: “Project”-“TCGA-SKCM”, “Data Category”-“clinical”; the CM RNA-seq: “Project”-“TCGA-SKCM”, “Data Category”-“transcriptome profiling”, “Data Type”-“Gene Expression Quantification”; the CM miRNA-seq: “Project”-“TCGA-SKCM”, “Data Category”-“transcriptome profiling”, “Data Type”-“Isoform Expression Quantification”. The miRcode database was used to obtain the relationship between differentially expressed miRNAs and lncRNAs. The target genes (mRNAs) of *hsa-miR-126-3p*, *hsa-miR-142-3p*, *hsa-miR-146b-5p*, *hsa-miR-17-5p*, *hsa-miR-22-3p*, *hsa-miR-23b-3p* and *hsa-miR-876-3p* were collected from the miRTarBase database, miRDB database and TargetScan database. The graph abstract of this study is shown in Fig. 5.

## Abbreviations

CM: Cutaneous melanoma
lncRNA: Long non-coding RNA
ceRNA: Competitive endogenous RNA
TCGA: The Cancer Genome Atlas
LASSO: Least absolute shrinkage and selection operator
ssGSEA: Single-sample gene set enrichment analysis
GO: Gene Ontology
KEGG: Kyoto Encyclopedia of Genes and Genomes
ROC: Receiver operating characteristic
AUC: Area under curve
